# Host species exploitation and discrimination by animal parasites

**DOI:** 10.1098/rstb.2016.0090

**Published:** 2017-03-13

**Authors:** Mark R. Forbes, André Morrill, Jennifer Schellinck

**Affiliations:** 1 Department of Biology, Carleton University, 1125 Colonel By Drive, Ottawa, Ontario, Canada K1S 5B6; 2 Institute of Cognitive Science, Carleton University, 1125 Colonel By Drive, Ottawa, Ontario, Canada K1S 5B6

**Keywords:** host species exploitation, host specialization, multi-host associations, parasite discrimination, population genetics

## Abstract

Parasite species often show differential fitness on different host species. We developed an equation-based model to explore conditions favouring host species exploitation and discrimination. In our model, diploid infective stages randomly encountered hosts of two species; the parasite's relative fitness in exploiting each host species, and its ability to discriminate between them, was determined by the parasite's genotype at two independent diallelic loci. Relative host species frequency determined allele frequencies at the exploitation locus, whereas differential fitness and combined host density determined frequency of discrimination alleles. The model predicts instances where populations contain mixes of discriminatory and non-discriminatory infective stages. Also, non-discriminatory parasites should evolve when differential fitness is low to moderate and when combined host densities are low, but not so low as to cause parasite extinction. A corollary is that parasite discrimination (and host-specificity) increases with higher combined host densities. Instances in nature where parasites fail to discriminate when differential fitness is extreme could be explained by one host species evolving resistance, following from earlier selection for parasite non-discrimination. Similar results overall were obtained for haploid extensions of the model. Our model emulates multi-host associations and has implications for understanding broadening of host species ranges by parasites.

This article is part of the themed issue ‘Opening the black box: re-examining the ecology and evolution of parasite transmission’.

## 1. Introduction

Many animal parasite species, like bat flies and fish monogeneans, are specialist parasites exploiting only one or a few host species [1,2]. In other species, such as diplostomatid flukes of fish, the parasites are specialists of particular tissues of their hosts, and metacercariae of single fluke species are again typically found in only one or a few host species [3]. This specificity of those fish flukes makes sense because they infect tissues where they are subject to host immunity: evasion of one host species' recognition mechanisms probably comes at the fitness cost of not being able to evade other host species' immune responses [3]. The fish eye flukes are an exception. Here, the eye of the fish has a blood barrier for immune reactions, which might otherwise blind the fish; as a result, these eye flukes tend to be generalist parasites [3].

Many other animal parasite species also appear as generalists, i.e. there are several or many host species exploited for a given life-history stage of the parasite [4]. Several studies have shown linkages between geographical range of a parasite species and its host species' range [5,6]. Importantly, this ability to exploit several to many host species occurs in many populations of animal parasites at single regions or sites. Furthermore, this multi-host species use or exploitation is found even after genetic tests for suites of cryptic parasite species. Broad generalist parasites are sometimes later revealed using molecular techniques as suites of cryptic species exploiting subsets of the available host species at single regions or sites, i.e. showing some degree of specialization. However, there are other instances where this is definitely not the case: generalist species remain generalist species following molecular analyses [7,8]. Further still, even for those tests where there are suites of cryptic species, the suites often include one or a few species that are still host species generalists (i.e. infect two or more host species) [9,10]. The same is true of other symbionts [11,12].

It is of particular interest from a micro-evolutionary perspective that individuals of a given parasite species that exploit two or more host species at a given site often show differential fitness on the different species of hosts. Remarkably, this differential fitness can include instances where the parasite has much lower, and even zero, fitness on one of those host species, but still attends to that host species [13,14]. This raises questions central to this study about why the parasite has not evolved host species discrimination (i.e. the capacity to detect and select for a host species upon which it will achieve high fitness). In some well-studied cases, the parasite is equally abundant on both susceptible and partially or wholly resistant species indicating little or no discrimination [13,15].

In explaining such non-discrimination, one might simply posit that infective stages of parasites lack the resources to discriminate. That is, selection is on parents to produce many offspring (e.g. cercariae of trematodes) and broadcast them in areas or at times when appropriate host species are present, even though mistakes are sometimes (or perhaps even frequently) made. We think this type of minimal allocation of resources to infective stages is an important reason why discrimination might be apparently lacking for certain parasite species. However, there are instances where no apparent discrimination is present even when there appears to be resources to discriminate [13,15].

Presumably, parasites would benefit from tracking abundant host species such that they become host specialists locally. But are such parasites local host specialists at the expense of using other host species that are more abundant in other regions or more abundant at other times? That is, do parasites trade off becoming local specialists with the likelihood of remaining global generalists (e.g. [16]; cf. [17])? This is really a question about whether genetic variation for host species use or exploitation will remain in parasite populations where one host species consistently outnumbers another, over a long enough period of time. Such questions are reminiscent of problems modelled in treatments of the geographical mosaic of coevolution [18]: here, this mosaic is applied to variable selection on parasite species across sites differing in the relative frequencies of host species. We can address the extent to which selection on parasites, at sites that are more suitable for one host species over another, either favours discrimination and/or loss of allelic diversity for host exploitation at those sites in the absence of gene flow. This is also reminiscent of disruptive selection on habitat or host preferences leading to host-race formation and sympatric speciation given sufficient reproductive isolation (cf. [19]).

Such changes can be modelled relatively easily if one is explicit about fitness trade-offs in using different host species (i.e. differential fitness), the relative host species frequencies in nature (which influence encounter rates of specific species of hosts) and the combined density of two or more host species (which influences encounter rate of any host and likely the cost of being discriminatory). Although other factors are likely also important, such as maternal resources allocated to infective stages of parasites, we concentrated on those three factors: differential fitness, relative host species frequency and combined host density.

In this paper, we describe an equation-based, two-locus model of host species use and discrimination. We refer hereafter to one locus as the exploitation locus and the other as the discrimination locus, each with two alleles. Throughout our paper, the response variables are the frequency of the alleles at either locus. We asked to what extent alleles for host species exploitation and host discrimination are maintained independently in parasite populations under varying conditions of overall host density, relative frequencies of different host species, and differences in fitness resulting from infecting either the host providing the highest fitness (superior host) or the alternative host. Such instances of animal parasites encountering two or more host species that differ in their suitability as hosts are expected to be common in nature.

## 2. Material and methods

All described simulation models were performed using R v. 3.0.2 [20]; an R script is included in the electronic supplementary material. We present an equation-based model following specific allele frequencies of a parasite population encountering two potential species of host. Our approach is very different from ‘gene-for-gene’ and also ‘matching allele’ approaches (reviewed in [21]), which are used when considering single species of parasites attacking or exploiting single species of hosts and where the genetics of each is considered or modelled specifically; here, we are only concerned with genetics of the parasite. It might seem odd to model non-evolving host species; however, we note that we are examining situations with strong host-mediated selection on parasites (parasites cannot complete their life cycle without hosts) and weaker parasite-mediated selection on hosts (e.g. the parasites are not main parasites of the host species being considered, or they exact low fitness costs to their hosts, and/or do not regulate their host populations). As mentioned, such occurrences are expected to be common in nature, although not considered explicitly in past studies.

**Figure 1.**
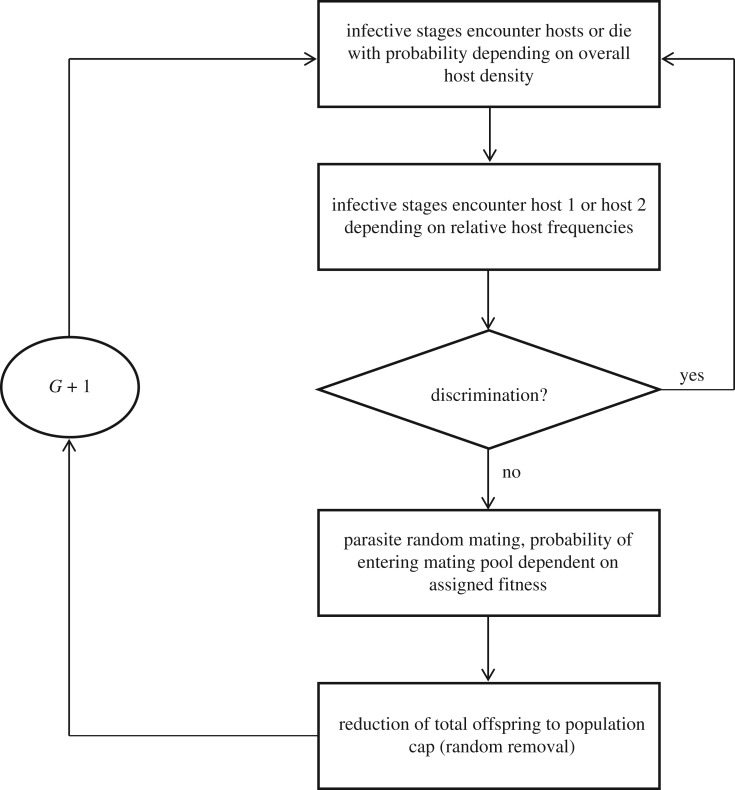
The conceptual framework of the parasite exploitation and discrimination model.

Parasites in our diploid model have greater fitness (modelled as probability of entering the mating pool) either on one or the other of the two potential host species, *h*_1_ or *h*_2_. Parasites are also either discriminatory or non-discriminatory (i.e. infective stages either re-enter or do not re-enter the search for another potential host after having encountered the alternative host species for which they would experience lower fitness). Each of these two characteristics, discriminatory ability and host exploitation potential, is coded at an independent diallelic locus. We model a system wherein infective stages search for a potential host, either encountering one or failing in their search (i.e. dying without locating a host). Those parasites that locate a host either discriminate or not, dependent on their discriminatory ability and the host species that is encountered (corresponding to lower or higher fitness, determined by alleles at the exploitation locus). Parasites then enter the mating pool with a probability dependent on their expectation of fitness from the infected host species. Mating and offspring production is simulated probabilistically following classical genetics of independent assortment of alleles from parents. Allele frequencies at both loci are tracked across generations. As mentioned, we then explore the effects of the following three factors on the evolution of discriminatory behaviour and host species exploitation among parasites: relative host frequencies (*h*_1_ versus *h*_2_), overall host density (relating to likelihood of infective stages encountering a host in any search event), and the difference in fitness between infecting the two host species. This modelled process is outlined in figure 1.

Our modelling of host species discrimination and exploitation by parasites coded at two separate loci is reminiscent of habitat choice and local adaptation models wherein habitat preference and relative performance are treated as two separate traits (cf. [22]). However, unlike previous models, we consider discriminatory ability of parasites to be ‘hard-wired’ and fully determined by the discrimination genotype, either occurring or not dependent on the identity of the encountered host and the exploitation genotype of the parasite. That is, discrimination is a binary response stemming from several interacting factors and not a coded probability (0 ≤ *p* ≤ 1) of remaining on a particular host (cf. [22]).

We also note that our modelling framework is adaptable to systems of plant selection by phytophagous insects. Unlike previous phytophagous insect models, we do not simulate host (plant) avoidance and attraction as genetically coded, but rather we model discrimination as host infection or abandonment after encounter [23].

The discriminatory locus consists of two possible alleles, *D* and *d*, determining discriminatory ability of the infective stage. Discriminatory parasites are presumed to discriminate (i.e. re-enter the host search stage) whenever they encounter the alternative host species for which they have lower fitness, therefore searching until they either encounter the other superior host species or ultimately fail to do so. We arbitrarily modelled the ability to discriminate (*D*) as dominant and non-discrimination (*d*) as recessive for our initial runs, but also ran all simulations with non-discrimination as the dominant allele for comparison. Dominance relations are in keeping with many genetic traits and their inclusion here ensures strong selection on discrimination.

The two alleles at the exploitation locus determine whether *h*_1_ (coded by allele *A*) or *h*_2_ (coded by *a*) is the host conferring the higher fitness for any given parasite (referred to before and hereafter as the ‘superior host’; the host which confers lower fitness we refer to as the ‘alternative host’). We could easily envision a situation where a parasite does less well on one host species because it is less able to evade that species' immune recognition (perhaps because it is predisposed to do well on another host species). Our model makes no stipulations about one host resisting more than another, although this might be the reason for low or no fitness on some host species, as seen in nature [13]. The *A* allele (*h*_1_ is superior) is set as dominant over *a*, though relative host frequencies (*h*_1_ versus *h*_2_ abundance) are modelled such that *h*_1_ is the common host species in some tested parameter sets and is the uncommon host species in other sets.

The model tracks the number of parasite individuals of each genotype from one generation to the next, then calculates allele frequencies at the two loci from those values. Given the total of four alleles at the exploitation and discrimination loci, there are nine possible genotypes, but only four phenotypes: *AADD*, *AADd*, *AaDD*, *AaDd* are all discriminatory and all experience greater fitness on *h*_1_; *AAdd* and *Aadd* produce phenotypes that do better on *h*_1_ and are non-discriminatory; *aaDD* and *aaDd* produce equivalent phenotypes that do better on *h*_2_ compared with *h*_1_, but are discriminatory; whereas *aadd* is the special case of a parasite that specializes on *h*_2_ and is non-discriminatory. The probability of any given genotype entering the mating pool is calculated with respect to the potential searching/discriminatory behaviour described above and potential host availability. This procedure is then expanded to determine the proportion of parasites of each genotype entering the mating pool. Offspring production and their corresponding genotypes were calculated based on the probabilities of individual mating pairs among parents (given the number of each parasite genotype in the mating pool) and the probability of each particular genotype being represented by offspring produced from those matings.

Proportions of each genotype entering the mating pool are calculated from their individual probabilities of successfully finding a host as infective stages and completing their life cycle. For non-discriminatory parasites (*dd*), this probability is simply the likelihood of encountering the superior host, added to the product of its likelihood of encountering the alternative host and the fitness of the parasite on that alternative host (the relative fitness of the parasite on the superior host is always equal to 1). This can be expressed as

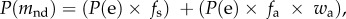
where *P*(*m*_nd_) is the probability of any non-discriminatory parasite entering the mating pool, *P*(e) is the probability of encountering any host (i.e. represented by the overall host density), *f*_s_ and *f*_a_ are the relative frequencies of the superior and alternative hosts respectively (*f*_s_ = 1 − *f*_a_), and *w*_a_ represents the fitness of the parasite on the alternative host. Whether *h*_1_ or *h*_2_ is the superior host for any given parasite depends on the alleles present at the exploitation locus.

Discriminatory parasites, on the other hand, cannot end their searching by contacting the alternative host, because upon encountering the alternative host discriminatory parasites would always re-initiate their search (i.e. discrimination is hard-wired). The probability of any discriminatory parasite entering the mating pool is equal to the probability of encountering the superior host in any single search relative to the combined probabilities of failing to find any host and a superior encounter in any single search:

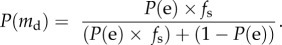


This is because each potential successive re-entry to the searching stage would contribute proportionally to the probabilities of the only two possible final outcomes (failure to find a host and infective stage death or encountering the superior host).

The proportion of each parasite genotype in the resulting offspring population in each generation is calculated as the sum of the probabilities of the offspring genotype of interest (for example, *g*_1_ of nine possible genotypes) being represented among offspring produced by a given mating pair of parents (mp), summed over all 45 possible mating pairs:

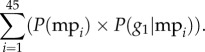


For mating pairs consisting of parents of the same genotype, the probability of the individual pairing (*P*(mp)) is calculated as the relative proportion of that genotype in the mating pool squared. For mating pairs consisting of two different parental genotypes, the probability is the product of the two relative proportions of individual genotypes in the mating pool, multiplied by two. In all simulations, the number of offspring resulting from each mating pair is set to 10.

While our main model simulates a diploid, sexually reproducing and semelparous parasite, we also ran modified simulations to consider asexual haploid parasites, wherein individuals successfully parasitizing hosts produce genetically identical offspring with a likelihood dictated by their fitness (determined by their exploitation locus allele(s) and the host they parasitize, i.e. equivalent to the probability of entering the mating pool in our main model). We compared these results to results of our main model to assess the influence of a mating system and dominance effects on final allele frequencies.

A given infective stage's probability of encountering a potential host individual depends on overall host density; if a parasite fails to find a host, it dies. This also determines the resulting cost of discrimination: given the ability to discriminate, a parasite which jumps from a host to which it is less adapted re-initiates host searching and again incurs the risk, dependent on overall host density, of not locating any host. We model the situation in which parasites attack two host species that coexist, but where the pairs of host species exist at different densities from other potential host species pairs. For example, imagine two related species of host water fleas at high combined density, versus two related species of host mosquito pupae at moderate combined density, and again versus host damselfly larvae at a lower combined density. We ask how the evolution of alleles at the exploitation and discrimination loci is affected by different degrees of host density interacting with relative host frequencies and the difference in fitness between infecting the superior versus alternative host (differential fitness). Again, we feel that these types of parasites (e.g. water flea parasites attacking other water flea species, but not species of mosquitoes) are reflective of nature (cf. [24]).

Exploring the evolution of host discrimination and changes in host exploitation by parasites resulted in many unique parameter combinations of the three factors tested; for each of these combinations, simulations were run for 100 generations, and results are averaged over 100 trials (despite being an equation-based model, random removal of parasites through the imposition of a population cap, described below, introduced a degree of stochasticity to our model). While 100 generations is a rather short time, we note that 100 generations (or 100 years for univoltine insects, for example) is a relatively long time for conditions to remain stable at a particular site (e.g. one host species remaining dominant over another). It is therefore a useful heuristic to focus our thinking on determinants of allelic variation. It is also important to note that 100 generations is not that short for profound change when selection is particularly strong. The magnitudes of differential fitness we modelled were very large, as are expected to be when parasites encounter potential alternative host species. Simulations were run with overall host density (i.e. encounter probability, *P*(e)) ranging from 0.1 to 0.9, in increments of 0.1. Relative host frequencies (*h*_1_ versus *h*_2_ abundances) similarly varied from 0.1 to 0.9 (ratios of 1 : 9, 2 : 8, 3 : 7 … 9 : 1). Finally, differential fitness varied from 1 (representing *w* = 1 on the superior host and *w* = 0, i.e. infective stage death, on the alternate host) to 0.1 (*w* = 1 on the superior host and *w* = 0.9 on the alternate host). Such variation in differential parasite fitness on different host species is expected, even for related species of hosts [25].

These combinations resulted in 9 host densities×9 relative host frequencies×10 differential fitness values = 810 parameter combinations. Initial allele frequencies (*A* and *a*; *D* and *d*) were always set to 50%, and alleles were distributed among the nine potential genotypes according to Hardy–Weinberg equilibrium with a starting parasite population of 1000. A population cap of 10 000 was imposed on the offspring pool at the end of each generation, with individuals being removed from each genotype randomly but with probability equal to that genotype's proportional representation in the population according to a multinomial distribution.

## 3. Results

**Figure 2.**
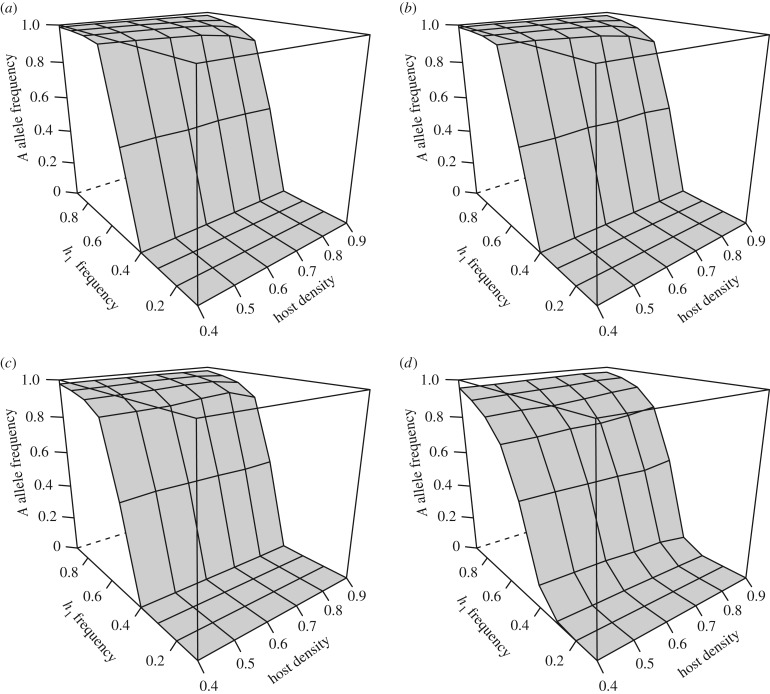
Final modelled exploitation allele (*A* allele) frequencies after 100 generations in the diploid sexual model given different relative host frequencies (*h*_1_ freq; ranging from 0.1 to 0.9) and overall host densities/probability of host encounter (*P*(e); 0.1 to 0.9), each parameter combination averaged over 100 trials. Panels (*a*–*d*) represent fitness differentials between the main and alternative host of 1.0, 0.7, 0.3 and 0.1, respectively.

The model demonstrates that when *h*_1_ and *h*_2_ are unequal in abundance, the exploitation allele (*A* or *a*) favouring the more common host species quickly comes to dominate the population (figure 2*a*–*d*; four of nine possible panels shown), and the speed at which it spreads through the population increases with increasing inequality between the two host frequencies (data not shown). Intermediate exploitation allele frequencies were observed after 100 generations only when average *h*_1_ frequency approximated 0.5. This trend of one allele becoming predominant or exclusively present was similar across all combined host densities when parasite populations did not crash. Extinctions were consistent at *P*(e) ≤ 0.2, while a *P*(e) = 0.4 was enough to guarantee population persistence at all differential fitness sets that were tested. The allele frequencies at the exploitation locus after 100 generations were largely, almost exclusively, affected by relative host frequencies (figure 2*a*–*d*). In any simulation where *h*_2_ frequency was higher, the *A* allele was completely purged from the host population; however, even though the *A* allele dominated in all trials where *h*_1_ was more common, the *a* allele was often still maintained in the population at a low proportion. This was certainly the result of it being masked by the dominant *A* allele in heterozygotes and hidden from selection (in the haploid asexual simulations, *a* alleles are most frequently lost when *h*_1_ hosts are more common; data not shown). As an example of masking, the average proportion of the *a* allele remaining in the sexual parasite population after 100 generations was 1.79% (±0.39%) at an overall host density of 0.8, even when *h*_1_ frequency was 0.9. Complete loss of genetic variation at this locus was only expected when the recessive allele *a* corresponded to exploitation of the more common host, with the dominant allele *A* going extinct. It is important to keep in mind that the results for the first locus (the exploitation locus) mean that, over most of the different parameter combinations, the populations consisted mostly if not exclusively of one allele (*A* or *a*) over another (except for the rare instance of host species frequencies remaining nearly equal).

**Figure 3.**
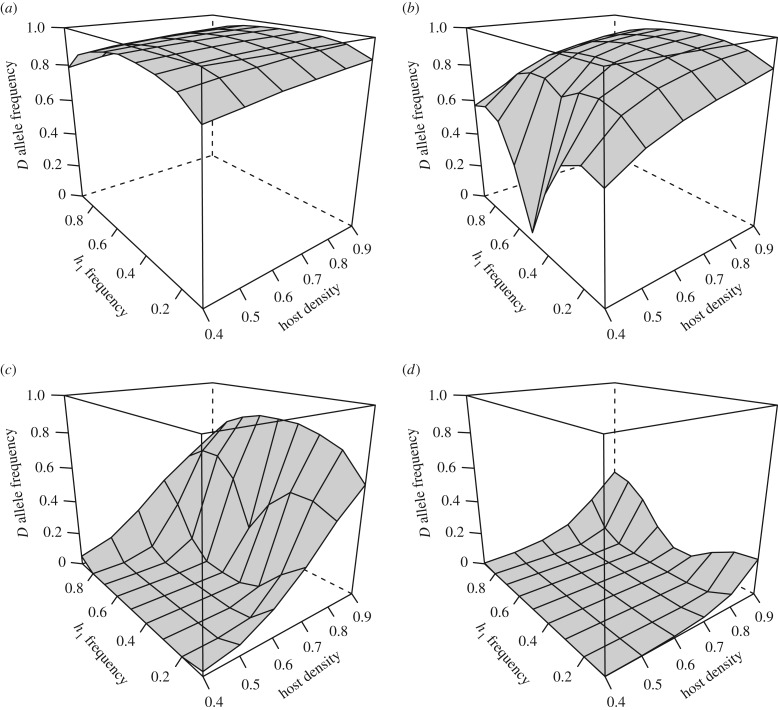
Final modelled discrimination allele (*D* allele) frequencies after 100 generations in the diploid sexual model given different relative host frequencies (*h*_1_ freq; ranging from 0.1 to 0.9) and overall host densities/probability of host encounter (*P*(e); 0.1 to 0.9), each parameter combination averaged over 100 trials. Panels (*a*–*d*) represent fitness differentials between the main and alternative host of 1.0, 0.7, 0.3 and 0.1, respectively.

The model has some interesting outputs with respect to the second locus of interest and the focus of this study. The degree to which the allele *D* (which favours discrimination to occur) is represented in the population versus the *d* allele, which in the homozygous recessive state (*dd*) confers non-discrimination, is affected principally by two factors (figure 3*a*–*d*). First, there is the differential fitness experienced between parasites on their superior versus alternative host species. Second, combined host density is important. For now, it is essential to recognize that discrimination could not be invoked unless the parasite had either one or two copies of the *D* allele. It is worth reiterating that discrimination was assumed to be ‘hard-wired’: parasites that landed on hosts to which they are less adapted would ‘jump’ to start searching again which could potentially (at least) lead to an increase in fitness for the individual.

When differential fitness is 1.0 (i.e. only parasitizing the main host can confer a fitness more than 0 and that fitness is equal to 1.0), discrimination always tends to evolve. Although generally not reaching fixation, the *D* allele tends to dominate under all simulated parameters (figure 3*a*). However, there is an important point to make. Even in this extreme case, there are some *d* alleles (never less than 2.4%, and as high as 21.3%) remaining in the parasite population; again, this is the result of the dominant *D* allele masking heterozygotes from selection against non-discrimination (similar to the exploitation locus when relative host frequencies are unbalanced, *d* alleles are frequently lost in the haploid asexual model when differential fitness is high; data not shown). Relative frequency of the *D* allele after 100 generations is slightly lowered when relative host frequencies are the most unbalanced (i.e. when either *h*_1_ or *h*_2_ makes up closer to 90% of the host population). This is because the exploitation allele (*A* or *a*) favouring the less common host is quickly reduced or even purged from the population. In these scenarios, parasites so seldom encounter the rare host that the selective pressure towards discrimination is decreased. Although host density did not strongly impact the fate of the discrimination locus alleles given this extreme differential fitness set, this lowering of *D* is somewhat more pronounced at lower overall host densities (increased risk during discrimination).

Given a differential fitness more than 0 but less than 1, a stronger influence of overall host density on discrimination allele frequencies emerges. When differential fitness is 0.7 (relative fitness of 0.3 on the alternative host), *D* allele frequency is still relatively high after 100 generations at higher (0.6–0.9) host densities, on average ranging from 72.4 to 96.4% (figure 3*b*). However, at combined host density of 0.4, non-discrimination is sometimes favoured, particularly at the most intermediate relative host frequencies (*h*_1_ versus *h*_2_, figure 3*b*), with average final *D* allele proportion ranging from 5.2 to 60.1%.

When the difference in resulting fitness between parasitizing the superior or alternative host was lower still (0.3), the alleles conferring non-discrimination (*d*) tended to dominate in all scenarios except those where overall host density was highest (figure 3*c*). Basically, there are many more instances in which the non-discriminatory allele *d* is favoured and often remains at very high frequencies. At lower overall host densities, when the risk of not finding a host when discriminating outweighs the minimal potential increase in fitness upon locating a superior host, the *D* allele was often purged from parasite populations, all individuals being non-discriminatory after 100 generations. It is interesting to note that in the majority of the (few) cases where allele frequencies at the discrimination locus after 100 generations were strongly affected by relative host frequency (e.g. differential fitness = 0.7, host density = 0.1; differential fitness = 0.3, host density = 0.8), it was when relative host frequencies were equal (*h*_1_ = *h*_2_ ∼ 50%), i.e. when neither *A* nor *a* came to dominate the host exploitation locus. This situation of both host species being consistently near equal in abundance over many generations is, however, unlikely in nature.

When differential fitness was relatively low at 0.1, non-discrimination was likely to evolve across a range of host densities (figure 3*d*). This result can be explained by the *D* allele not being favoured when the fitness expectation of first encountering the alternative host species is still relatively high.

We also ran models with the non-discriminatory allele as dominant to compare the effect on the maintenance of non-discrimination among parasites. While the results were qualitatively similar, in cases where the allele coding for discrimination only approached fixation before, it achieved fixation with the dominance reversed; conversely, alleles coding for non-discrimination were largely prevented from reaching fixation. However, it is important to note that in both tested dominance relationships at the discrimination locus there were a variety of parameter combinations where both the *D* and *d* alleles persisted in the population through the simulations even without one or the other approaching fixation (i.e. tested populations of parasites could persist with intermediate proportions of both the *D* and *d* alleles).

The simulations involving asexually reproducing parasites produced qualitatively similar results compared to the main models: precisely the same parameter combinations led to either increased or decreased allele frequencies at both the exploitation and discrimination loci, with the influence of dominance effects as the sole dissimilarity. Not surprisingly, in the haploid model, recessive alleles (either *a* or *d*) could not be masked from selection in heterozygotes and were therefore completely purged from populations wherein they would otherwise have been maintained at low frequencies in the diploid sexual model.

## 4. Discussion

There is considerable evidence that animal parasites of single species are able to exploit several to many host species and have differential fitness on different host species [26]. So when should parasites be discriminatory? This is equivalent to asking why parasites still fail to discriminate against host species where they experience lower, and sometimes even zero, fitness (either in or on hosts). One general argument is that some fitness is better than no fitness; if the parasites are too discriminatory they might not get any host opportunities (there are likely costs of discrimination). Our model shows that this point can be moot at the lowest combined host density. If the combined host density is too low, the parasite population can go extinct. While the data were not shown here, all parasite populations went extinct at the lowest combined host density (0.2) that we considered. Another way to state this important finding is that host density can become so low as to result in extinction before it has the opportunity to favour non-discrimination.

It is therefore not just a question of low encounter probability causing parasites to evolve non-discrimination. A combined host density of 0.4 (still quite low, but high enough for the parasite population to keep from extinction after 100 generations) resulted in populations that are either principally discriminatory or non-discriminatory depending on whether differential fitness is high or low, respectively.

Beyond the special case of lowest combined host density, it is really the interplay of combined host density, differential fitness and relative host frequency that was expected to determine the fate of alleles at both the exploitation and discrimination loci. Here, our model has several salient findings. First, as expected, relative host frequency is by far the most important determinant of allelic frequency at the exploitation locus. As relative host frequencies become unbalanced (across all differential fitness sets, four of nine shown in figure 2*a–d*), one allele comes to dominate if not exclude the other allele in the population, across all combined host densities. The abruptness with which this occurs is slightly dampened when differential fitness is lower (0.1; figure 2*d*), but the end result is the same. Populations are either made up principally or exclusively of *A* or *a* (except in the unlikely instance where relative host frequencies consistently hover around 50 : 50)*.* This leads to the interesting observation that allelic diversity for exploitation is only maintained under such strong selection when the dominant allele confers greater fitness to the common host. It is also worth mentioning that evolution at the exploitation locus occurs independently of evolution at the discrimination locus. For example, evolution of populations that are entirely discriminatory or non-discriminatory will have either *A* or *a* dominate depending on relative host frequency only.

At the discrimination locus, it is really the differential fitness that is important. At high differential fitness, discrimination is likely to evolve over a range of combined host densities. Remarkably, there are cases in nature where the parasites have zero fitness on alternative host species and yet still occur on those hosts [13,15]. These types of patterns are important and interlinked. In our model, we are starting with differential fitness on different hosts and observing under which conditions discrimination can still be selected against. We could also start with parasites being non-discriminatory and see under what conditions the hosts evolve resistance to the parasites (but here, the host genetics would be treated explicitly; see [14] for examples of such tests). It is quite possible that the finding in nature of hosts totally resistant to non-discriminatory parasites is due to two processes operating in step fashion over coevolutionary time, i.e. the evolution of non-discrimination by parasites followed by the evolution of one species' host resistance to parasites. The latter point has recently been suggested to be a result of some host species being in closed populations and evolving parasite recognition that is not countered by selection on the generalist parasite (despite the asymmetry of stronger selection on parasites compared with their hosts that is often invoked; reviewed in [14]).

We might also expect in nature that parasites that attack certain host species are able to attack related host species and show low differential fitness on both, at least initially. Our lowest value of differential fitness of 0.1 is not that low at all, and it is worth considering even lower values (e.g. 0.01), at least initially. That would set up the context for the evolution of non-discrimination over varying host densities (figure 3*d*), although as higher differential fitness creeps in (selection imposed by hosts) there is only selection for non-discrimination at the lowest combined host densities (compare figure 3*c*,*d*). Two extensions of the model are worth noting. The first is that we might expect discrimination when combined densities of hosts are highest, providing there is moderate to high differential fitness. This is somewhat equivalent to suggesting more abundant organisms and their related species are more likely to have specialist parasites. Less abundant species and their related species might be more likely to have non-discriminatory parasites because combined host densities are lower and this plays out as a cost to being discriminatory. To our knowledge, this suggestion of host abundance influencing degree to which parasites are generalist or specialist has not been tested. It might, however, help explain scenarios where high host-specificity is found alongside high effective host density, although seemingly counter to the other theory [1].

The second extension is that we might often expect mixes of discriminatory and non-discriminatory parasites in single populations. Tests could be easily conceived but difficult to execute to explore whether there is genetic basis to being discriminatory and how selection operates on such a trait under varying conditions of combined host density and relative host frequency. Regardless, this leads to the expectation that a mix of discrimination and non-discrimination might be present in populations and set the stage for host-race formation (see [27]). It will of course be important to consider parasite attributes in such tests. For example, host use will depend on realized host density, which depends on parasite mobility and ability to search actively for hosts. Within species, we might expect cases where less mobile stages are actually less discriminatory (see [28], where larval ticks are less ambulatory, and less discriminatory, than adults).

As indicated earlier, it is important to bear in mind that selection might be stronger on adults rather than infective stages. Adults could produce many young that are weakly active or not good at searching. It might be a mistake to consider infective stages as principally the individuals under selection, but rather as collectives of individuals produced by adults (under selection) who put infective stages in the host's way. Adults might not invest much in infective stages (‘win some by producing many’ problem). In such cases, infective stages might not have the resources to be discriminatory in the first place. However, there is evidence that parasite infective stages are discriminatory in other groups such as larval mites attacking pupal mosquitoes, presumably when it is advantageous to do so [29]. Whether adults of any animal parasites follow a diversification strategy (cf*.* [30]) by investing variably in offspring in ways that affect the expression of discrimination remains to be seen.

The presented model and findings may be most relevant for associations involving ectoparasites or those endoparasites that gain entry to the host actively. This is because the model presumes some agency on the part of the infective stage to potentially accept or abandon the encountered host. Trophically transmitted endoparasites have little means or opportunity to discriminate between hosts, and the model might not be relevant to them. Compare this with strepsipteran parasites, for example, which encounter and enter their insect hosts through the host cuticle as infective first instar larvae: the females (except those from the family Mengenillidae) inhabit as endoparasites for the remainder of their lives [10].

Our model is also relevant for phytophagous insect–plant systems. Here, plant choice and specificity is worth considering: insofar as instances of discrimination between plants can carry potential fitness consequences, insects should be less discriminatory, and less specific, when abundances of potentially exploited plants are low. Of course, alternative explanations will have to be considered, such as poor food plants being exploited because they provide protection from parasitoids.

The models described in this study specifically simulate host discrimination by parasites after an initial host encounter, as opposed to modelling discriminatory behaviour that would either increase or decrease the probability of host contact in response to potential host cues at a distance. This post-encounter type of discrimination is seen in experiments. For example, Smith & McIver [29] observed that two species of larval *Arrenurus* water mites were equally attracted to four potential *Aedes* spp. host mosquitoes, but after physical encounter the mites were far less likely to remain in association with two of the four. This experiment is particularly interesting because it shows a clear differentiation between pre- and post-encounter host species recognition mechanisms, and the importance of the latter in host discrimination. Egan [31] demonstrated that *Proctolaelaps nauphoetae*, parasitic mites of the cockroach *Nauphoeta cinerea*, use both chemical cues specific to their host and mechanical stimuli, both detected exclusively at host contact, when discriminating potential hosts. Also, the ability to make parasitism choices based upon characteristics detected at the encounter of a potential host have been described among parasitoid wasps, for example to avoid or promote superparasitism [32–34], or even to promote the laying of multiple eggs given the presence of a protective bacterial symbiont that decreases parasitoid egg survival [35].

Perhaps not surprisingly, the majority of studies considering host preference behaviour examine factors contributing to host locating and/or avoidance in response to different cues, i.e. parasite discrimination of potential hosts prior to (though affecting the probability of) host encounter. For example, parasites can use host-related chemoreceptory and olfactory cues (e.g. [31,36,37]), light [38], vibrations [39], heat [40], carbon-dioxide [37,40], and, among some aquatic parasites, changes in current [41] to help locate (or avoid) certain potential hosts. Even in cases where parasites respond to host-related cues prior to host contact, it is still expected that additional host discrimination should occur after contact with the potential host, some final host-specific cues being required for the decision to parasitize to be initiated [40,41]. Importantly, if the only discrimination is pre-encounter, then our model and its results are still applicable. Experiments with trematode larvae suggest some form of pre-encounter discriminatory behaviour related to the probability of successfully infecting a host [42].

One final note concerns the importance of the retention of *d* alleles if the parasite invades a new site with a suite of different host species. This model can be expanded to include factors favouring the maintenance of spatio-temporal variation in allele frequencies. The *d* alleles in the homozygous recessive form will ensure some individuals ‘exploit’ new host species indiscriminately and those parasites might also have the alleles favouring use of that host species. In this way, we might expect to see local specialists evolving into global generalists depending on the degree to which host species change or change in abundance from site to site (or within sites over time). In the case of parasites in or on hosts, non-discrimination might prove useful in terms of evolutionary potential for broadening the host species range, but it is difficult to see how selection would favour this broadening, except as a consequence of selection on other traits (non-discrimination evolves because discrimination is not very advantageous in certain situations). Of course, there are many other factors (e.g. host innate immunity) that will dictate whether a new functional parasite–host association is created and whether this broadening of host species range results in further ecological or evolutionary change.

## Supplementary Material

Main model - R script

## References

[1] Dick CW, Patterson BD 2007 Against all odds: explaining high host specificity in dispersal-prone parasites. Int. J. Parasitol. 37, 871–876. (10.1016/j.ijpara.2007.02.004) 17382332

[2] Poulin R 1992 Determinants of host-specificity in freshwater fishes. Int. J. Parasitol. 22, 753–758. (10.1016/0020-7519(92)90124-4) 1428509

[3] Locke SA, McLaughlin JD, Dayanandan S, Marcogliese DJ 2010 Diversity and specificity in *Diplostomum* spp. metacercariae in freshwater fishes revealed by cytochrome *c* oxidase I and internal transcribed spacer sequences. Int. J. Parasitol. 40, 333–343. (10.1016/j.ijpara.2009.08.012) 19737570

[4] Lajeunesse MJ, Forbes MR 2002 Host range and local parasite adaptation. Proc. R. Soc. Lond. B 269, 703–710. (10.1098/rspb.2001.1943) PMC1690949 11934361

[5] Szöllősi E et al. 2011 Determinants of distribution and prevalence of avian malaria in blue tit populations across Europe: separating host and parasite effects. J. Evol. Biol. 24, 2014–2024. (10.1111/j.1420-9101.2011.02339.x) 21726328

[6] Mlynarek J, Knee W, Smith B, Forbes M 2015 Regionally widespread parasitic water mites have relatively broad host species ranges. Can. J. Zool. 93, 741–746. (10.1139/cjz-2015-0077)

[7] Mlynarek J, Knee W, Forbes M 2014 Host phenology, geographic range size and regional occurrence explain interspecific variation in damselfly–water mite associations. Ecography 37, 670–680. (10.1111/ecog.00997)

[8] Goulding TC, Cohen CS 2014 Phylogeography of a marine acanthocephalan: lack of cryptic diversity in a cosmopolitan parasite of mole crabs. J. Biogeogr. 41, 965–976. (10.1111/jbi.12260)

[9] Smith MA, Wood DM, Janzen DH, Hallwachs W, Hebert PDN 2007 DNA barcodes affirm that 16 species of apparently generalist tropical parasitoid flies (Diptera, Tachinidae) are not all generalists. Proc. Natl Acad. Sci. USA 104, 4967–4972. (10.1073/pnas.0700050104) 17360352 PMC1821123

[10] Jůzová K, Nakase Y, Straka J 2015 Host specialization and species diversity in the genus *Stylops* (Strepsiptera: Stylopidae), revealed by molecular phylogenetic analysis. Zool. J. Linn. Soc. 174, 228–243. (10.1111/zoj.12233)

[11] Knee W, Beaulieu F, Skevington JH, Kelso S, Cognato AI, Forbes MR 2012 Species boundaries and host range of tortoise mites (Uropodoidea) phoretic on bark beetles (Scolytinae), using morphometric and molecular markers. PLoS ONE 7, e47243 (10.1371/journal.pone.0047243) 23071768 PMC3469529

[12] Knee W, Beaulieu B, Skevington JH, Kelso S, Forbes MR 2012 Cryptic species of mites (Uropodoidea: *Uroobovella* spp.) associated with burying beetles (Silphidae: *Nicrophorus*): the collapse of a host generalist revealed by molecular and morphological analyses. Mol. Phylogenet. Evol. 65, 276–286. (10.1016/j.ympev.2012.06.013) 22732596

[13] Mlynarek JJ, Knee W, Forbes MR 2014 Explaining susceptibility and resistance to a multi-host parasite. Evol. Biol. 41, 115–122. (10.1007/s11692-013-9251-6)

[14] Forbes MR, Mlynarek JJ 2014 A hypothesis to explain host species differences in resistance to multi-host parasites. Ideas Ecol. Evol. 7, 17–24. (10.4033/iee.2014.7.5.n)

[15] Forbes MR, Muma K, Smith BP 1999 Parasitism of *Sympetrum* dragonflies by *Arrenurus planus* mites: maintenance of resistance particular to one species. Int. J. Parasitol. 29, 991–999. (10.1016/S0020-7519(99)00061-2) 10501609

[16] Knee W, Forbes MR, Beaulieu F 2013 Diversity and host use of mites (Acari: Mesostigmata, Oribatida) phoretic on bark beetles (Coleoptera: Scolytinae): global generalists, local specialists? Annu. *Entomol. Soc. Am.* 106, 339–350. (10.1603/an12092)

[17] McCoy KD, Léger E, Dietrich M 2013 Host specialization in ticks and transmission of tick-borne diseases: a review. Front. Cell. Infect. Microbiol. 3, 57 (10.3389/fcimb.2013.00057) 24109592 PMC3790072

[18] Thompson JN 2005 The geographic mosaic of coevolution. Chicago, IL: University of Chicago Press.

[19] Rice WR 1987 Speciation via habitat specialization: the evolution of reproductive isolation as a correlated character. Evol. Ecol. 1, 301–314. (10.1007/BF02071555)

[20] R Development Core Team. 2013 R: A language and environment for statistical computing. Vienna, Austria: R Foundation or Statistical Computing See http://www.R-project.org/

[21] Schmid-Hempel P 2011 Evolutionary parasitology: the integrated study of infections, immunology, ecology, and genetics. Oxford, UK: Oxford University Press.

[22] Ravigné V, Dieckmann U, Olivieri I 2009 Live where you thrive: joint evolution of habitat choice and local adaptation facilitates specialization and promotes diversity. Am. Nat. 174, E141–E169. (10.1086/605369) 19737113

[23] Feder JL, Forbes AA 2007 Habitat avoidance and speciation for phytophagous insect specialists. Funct. Ecol. 21, 585–597. (10.1111/j.1365-2435.2007.01232.x)

[24] Hadfield JD, Krasnov BR, Poulin R, Nakagawa S 2014 A tale of two phylogenies: comparative analyses of ecological interactions. Am. Nat. 183, 174–187. (10.1086/674445) 24464193

[25] Mlynarek JJ, Iserbyt A, Nagel L, Forbes MR, Söderhäll K 2015 Differential water mite parasitism, phenoloxidase activity, and resistance to mites are unrelated across pairs of related damselfly species. PLoS ONE 10, e0115539 (10.1371/journal.pone.0115539) 25658982 PMC4319886

[26] Bielby J, Fisher MC, Clare FC, Rosa GM, Garner TWJ 2015 Host species vary in infection probability, sub-lethal effects, and costs of immune response when exposed to an amphibian parasite. Sci. Rep. 5, 10828 (10.1038/srep10828) 26022346 PMC4448222

[27] Magalhães S, Forbes MR, Skoracka A, Osakabe M, Chevillon C, McCoy KD 2007 Host race formation in the Acari. Exp. Appl. Acarol. 42, 225–238. (10.1007/s10493-007-9091-0) 17674128

[28] Dietrich M, Lobato E, Boulinier T, McCoy KD 2014 An experimental test of host specialization in a ubiquitous polar ectoparasite: a role for adaptation? J. Anim. Ecol. 83, 576–587. (10.1111/1365-2656.12170) 24467400

[29] Smith BP, McIver SB 1984 Factors influencing host selection and successful parasitism of *Aeda*. Can. J. Zool. 62, 1114–1120. (10.1139/z84-162)

[30] Simons AM 2009 Fluctuating natural selection accounts for the evolution of diversification bet hedging. Proc. R. Soc. B 276, 1987–1992. (10.1098/rspb.2008.1920) PMC2677257 19324774

[31] Egan ME 1976 The chemosensory bases of host discrimination in a parasitic mite. J. Comp. Physiol. 109, 69–89. (10.1007/BF00663436)

[32] Bakker K, van Alphen JJM, van Batenburg FHD, van der Hoeven N, Nell HW, van Strien-van Liempt WTFH, Turlings TCJ 1985 The function of host discrimination and superparasitization in parasitoids. Oecologia 67, 572–576. (10.1007/BF00790029) 28311043

[33] van Alphen JJM, Visser ME 1990 Superparasitism as an adaptive strategy for insect parasitoids. Annu. Rev. Entomol. 35, 59–79. (10.1146/annurev.en.35.010190.000423) 2405774

[34] Le Ralec A, Anselme C, Outreman Y, Poirié M, van Baaren J, Le Lann C, van Alphen JJM 2010 Evolutionary ecology of the interactions between aphids and their parasitoids. C. R. Biol. 333, 554–565. (10.1016/j.crvi.2010.03.010) 20541166

[35] Oliver KM, Noge K, Huang EM, Campos JM, Becerra JX, Hunter MS 2012 Parasitic wasp responses to symbiont-based defense in aphids. BMC Biol. 10, 11 (10.1186/1741-7007-10-11) 22364271 PMC3312838

[36] Krasnov BR, Khokhlova IS, Oguzoglu I, Burdelova NV 2002 Host discrimination by two desert fleas using an odour cue. Anim. Behav. 64, 33–40. (10.1006/anbe.2002.3030)

[37] Dillman AR, Guillermin ML, Lee JH, Kim B, Sternberg PW, Hallem EA 2012 Olfaction shapes host–parasite interactions in parasitic nematodes. Proc. Natl Acad. Sci. USA 109, E2324–E2333. (10.1073/pnas.1211436109) 22851767 PMC3435218

[38] Mordue (Luntz) AJ, Birkett MA 2009 A review of host finding behaviour in the parasitic sea louse, *Lepeophtheirus salmonis* (Caligidae: Copepoda). J. Fish Dis. 32, 3–13. (10.1111/j.1365-2761.2008.01004.x) 19245627

[39] Lawrence PO 1981 Host vibration–a cue to host location by the parasite, *Biosteres longicaudatus*. Oecologia 48, 249–251. (10.1007/BF00347971) 28309807

[40] Lourenço SI, Palmeirim JM 2008 How do ectoparasitic nycteribiids locate their bat hosts? Parasitology 135, 1205–1213. (10.1017/S003118200800468X) 18664305

[41] Boxshall GA 1976 The host specificity of *Lepeophtheirus pectoralis* (Müller, 1776) (Copepoda: Caligidae). J. Fish Biol. 8, 255–264. (10.1111/j.1095-8649.1976.tb03949.x) 4419475

[42] Sears BF, Schlunk AD, Rohr JR 2012 Do parasitic trematode cercariae demonstrate a preference for susceptible host species? PLoS ONE 7, e51012 (10.1371/journal.pone.0051012) 23272084 PMC3525650

